# AESTHETIC PATHWAYS IN GEROTRANSCENDENCE

**DOI:** 10.1093/geroni/igae098.1980

**Published:** 2024-12-31

**Authors:** Sarah McKiddy

**Affiliations:** University of Washington, Seattle, Washington, United States

## Abstract

This presentation aims to enrich and expand the discourse on aging by exploring overaps and areas for exploration within Tornstam’s theory of gerotranscendence and the tenets of Rowe & Kahn’s successful aging model, with a focus on aesthetics as a mechanism for exploring and expressing the aging process. Gerotranscendence involves a shift in cognitive and emotional paradigms, marked by a reevaluation of purpose, interconnectedness, and self-awareness. Gerotranscendence augments the potential for well-being and health promotion by cultivating increased and meaningful engagement with the world and an evolution in perceptions and consciousness as individuals age. Encompassing diverse artistic expressions such as visual arts, music, and creative activities, aesthetics provide an individualized medium for introversion and self-expression. This multipronged approach to aging considers how individuals navigate senescence and nurture a connection with their inner selves alongside chronic conditions, diseases, limitations, or age-related changes. Engaging in aesthetic experiences offers depth and cognitive consonance in addressing feelings of loss, nostalgia, or existential questions related to aging. Aesthetics, then, emerge as a compelling conduit to convey the transitions and shifts described in the process of gerotranscendence. Integrating gerotranscendence and aesthetics within the context of traditional aging notions both illuminates and deepens the understanding of successful aging by accentuating the psychological, emotional, and creative dimensions, promoting a more holistic and arguably more incisive perspective on aging.

